# Morphological Methods to Evaluate Peripheral Nerve Fiber Regeneration: A Comprehensive Review

**DOI:** 10.3390/ijms24031818

**Published:** 2023-01-17

**Authors:** Giulia Ronchi, Federica Fregnan, Luisa Muratori, Giovanna Gambarotta, Stefania Raimondo

**Affiliations:** Department of Clinical and Biological Sciences & Neuroscience Institute Cavalieri Ottolenghi (NICO), University of Torino, Orbassano, 10043 Torino, TO, Italy

**Keywords:** peripheral nerve regeneration, morphological outcome measurement, measurement reliability and reproducibility

## Abstract

Regeneration of damaged peripheral nerves remains one of the main challenges of neurosurgery and regenerative medicine, a nerve functionality is rarely restored, especially after severe injuries. Researchers are constantly looking for innovative strategies for tackling this problem, with the development of advanced tissue-engineered nerve conduits and new pharmacological and physical interventions, with the aim of improving patients’ life quality. Different evaluation methods can be used to study the effectiveness of a new treatment, including functional tests, morphological assessment of regenerated nerve fibers and biomolecular analyses of key factors necessary for good regeneration. The number and diversity of protocols and methods, as well as the availability of innovative technologies which are used to assess nerve regeneration after experimental interventions, often makes it difficult to compare results obtained in different labs. The purpose of the current review is to describe the main morphological approaches used to evaluate the degree of nerve fiber regeneration in terms of their usefulness and limitations.

## 1. Introduction

Peripheral nerves are spread throughout the body and are highly vulnerable to injuries. Therefore, nerve injuries are extremely common, affecting, globally, over one million people every year [1]. Although usually not threatening to the patient’s life, such injuries affect physical capabilities due to loss of motor or sensory functions, and also have a significant effect on psychosocial aspects, diminishing the patient’s life quality [2].Traumatic nerve injury results in fragmentation of the distal axon and loss of synaptic terminals in a process known as Wallerian degeneration. Consequently, the target skeletal muscle enters into a state of inaction triggering muscular atrophy. Ultimately, the nerve may regenerate and restore a functional connection with target muscles.

Despite advancement in reconstructive microsurgery and tissue engineering, recovery of motor and sensory function after injury is suboptimal [3,4,5]. Alternative treatment or easy-to-apply supportive strategies for stimulating nerve growth and functional recovery should therefore be sought.

The proper assessment of the degree of nerve regeneration is essential to determine the effects of a new drug, treatment or strategy in improving nerve regeneration after injury and the selection of adequate evaluation methods is a key point to provide the appropriate information.

A method is suitable if it is helpful in elucidating the events occurring in the process of nerve regeneration and if it allows us to compare different strategies used to repair the damaged nerve and to improve nerve regeneration in order to find the best ones. Usually, there is no single method that is preferable to others; rather, more methods are combined to answer specific scientific questions.

Different assessment categories are currently used by researchers for the evaluation of peripheral nerve regeneration after trauma and nerve reconstruction: behavioral recovery by means of motor and sensory tests, evaluation of reinnervated target muscles through electrophysiological measurements together with the analysis of reinnervated muscle fibers, histomorphometry and immunostaining analysis of regenerated nerve fibers (both myelinated and unmyelinated), nodal and internodal length and analysis of neuron cell bodies in the spinal cord and dorsal root ganglia. An overview of functional evaluation methods and analyses of the reinnervation of target organs have already been reviewed elsewhere [6,7,8,9].

This review provides an integrative and critical overview of the most used and useful methods that have been employed to evaluate the morphological outcome measurements of nerve regeneration following injury and repair in experimental animal models with a specific focus on regenerated nerve fibers.

## 2. General Concepts

The choice of the experimental animal model (i.e., mouse, rat, rabbit, sheep, monkey) and nerve model (mixed, purely motor or purely sensitive nerve) is essential and depends on the aim of the study. Most studies on peripheral nerve regeneration are carried out using the sciatic nerve of rodents [10], although other animal and nerve models can be employed [9,11].

Moreover, the type of nerve injury/repair (axonotmesis or neurotmesis with or without substance loss), the lesion site (more proximal or distal to the target organs), the timing (immediate or delayed repair), the age and, possibly, the gender, are effective elements that must be taken into consideration when studying nerve regeneration, because each one can influence the speed and degree of the regenerative process and therefore the morphological/functional outcomes.

In addition, when sampling peripheral nerves for morphological analysis, considerable care must be given to select the appropriate methodology (fixative solutions, tissue orientation, embedding and cutting procedures and staining protocols) to ensure that tissue morphology is optimally preserved and to apply the correct morphological analysis [12,13,14].

## 3. Morphological Methods and Myelinated Fiber Quantification

The number of nerve fibers is considered a good indicator by which to compare the efficacy of different strategies, but to explain the significance of the number that is obtained after the counting, these data must be considered together with size parameters.

An unchanged number of nerve fibers of equal size compared with a normal nerve can represent the most favorable situation; however, it is known that regenerating fibers branch, that they increase in number distally to the lesion site and that they start growth as very small fibers that increase in diameter during regeneration. After long-time regeneration, the number of nerve fibers could return to normal values, but both axon and fiber diameter and myelin thickness remain significantly lower than in a healthy nerve [15].

Considering the size parameters, it is possible to define the g-ratio—the ratio between axon and fiber diameter. This is a very important value for the evaluation of the axonal function recovery during regeneration since it is correlated with the maturation of fibers and their speed of conduction [16].

The quantitative analysis of regenerating fibers is normally performed in a single 2D cross-section of the nerve. For this reason, and since nerve fibers sprout during regeneration, in order to allow a comparison between different nerves, it is very important to perform the analysis at the same distance from the point of lesion.

Unfortunately, the wide variety in methods used in literature for myelinated fiber counting makes the comparison of published data difficult to deal with. In addition, the different methods used to process the nerves (e.g., resin or paraffin embedding), the type of staining (haematoxylin and eosin, Trichrome, osmium, Toluidine Blue) or the quality of the microscope and camera used for the analysis could bias the counting results [12].

**Figure 1:**
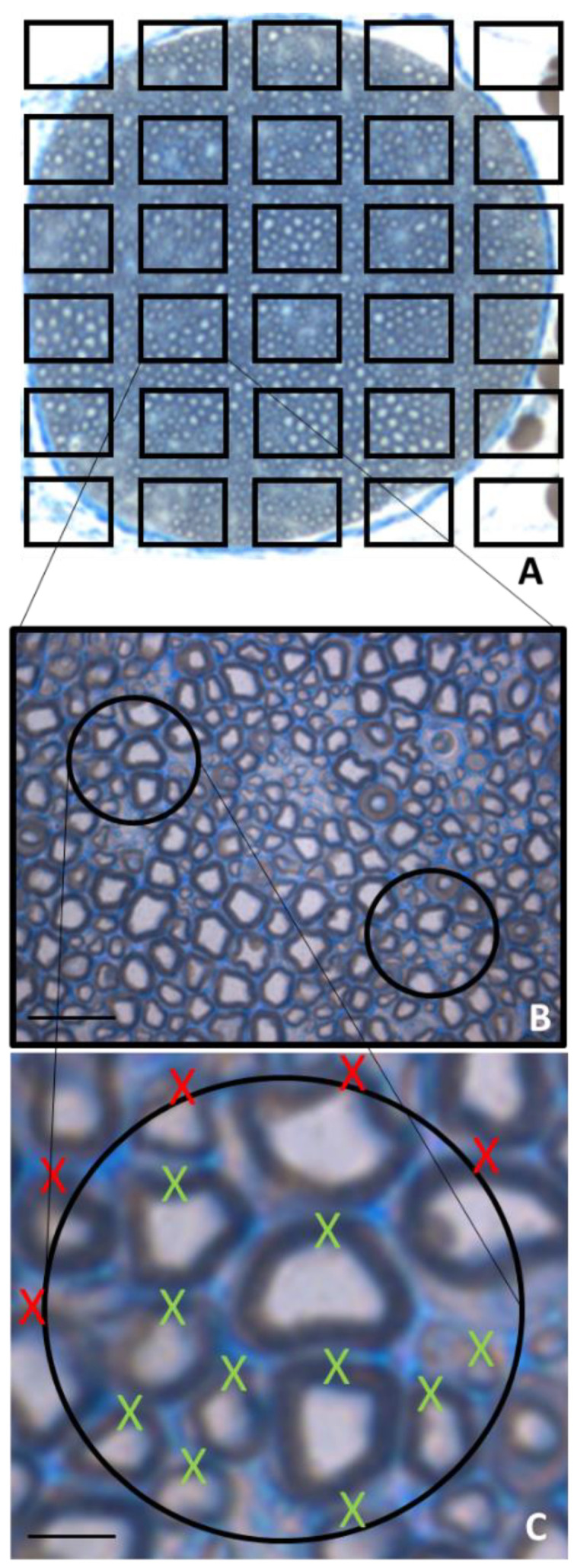
Representative scheme of morphometric procedures for the quantitative analysis of myelinated nerve fibers on a semithin transvers section stained with toluidine blue. (**A**,**B**) Systematic random sampling rules representation; (**C**) 2D dissector method based on sampling the “tops” of fibers, avoiding the “edge effect”. X red: fiber not counted. X green: fiber counted. Scale bars: 20 μm (**B**); 5 μm (**C**).

Full manual, sampled manual and sampled automatic count methods are the most frequently used methods for counting myelinated fibers. Recently, Orfahli et al., demonstrated a significant difference among results obtained using the full manual method and the other two [17]. Full manual count is performed on the full nerve cross-section and for this reason is time-consuming and subject to human error [18]; therefore it is rarely used, while sampled manual method remains the most accurate alternative. Among the various technical issues associated with a good morphoquantitative analysis, sampling strategy plays a critical role. Unfortunately, many studies reporting data on peripheral nerve counting, contain either methodological sampling errors or, more commonly, insufficient methodological information for a clear evaluation of the significance of the results. To ensure that every part of the nerve cross-section has the same probability of being included in the sample, systematic random sampling rules have to be followed. Moreover, the 2D dissector method enables us to determine the number of fibers in a known area independent from their size and shape since it is based on sampling the “tops” of fibers in order to avoid the “edge effect” (Figure 1) [19]. Furthermore, researchers should be well-trained; sometimes, it is not easy to discriminate a myelinated fiber from other structures present in the nerve tissue, especially during regenerative conditions. It is also important that the same researcher takes care of quantifying all the samples of a certain study (possibly by carrying out “blind” analyses) to avoid the accumulation of errors due to the involvement of several researchers.

In most studies [20,21,22,23], automated analysis of a nerve cross-section is performed using different software (e.g., Image Pro Plus; AxonJ plugin of ImageJ; AxonDeepSeg). Automated counting is the most time efficient, but involving almost no manual intervention, its potential for error is well-known. In fact, automated programs can fail to recognize nuclei, blood vessels, axons, distorted structures and artefacts. To compensate for possible errors in counting, the combination of manual and automatic methods, meaning semiautomatic analysis, is now one of the most common approaches for peripheral nerve morphometry [24].

**Figure 2:**
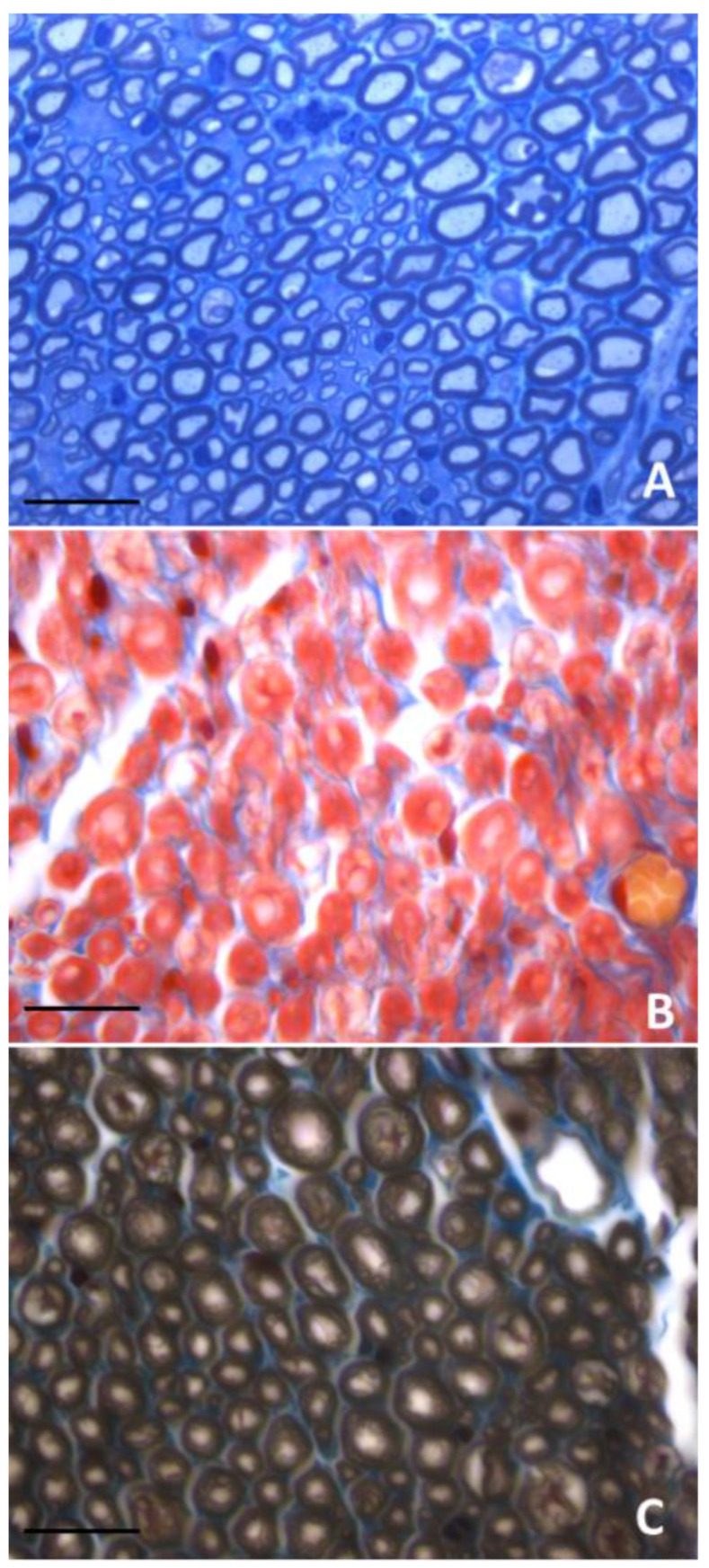
Transverse sections of healthy peripheral nerves subjected to different tissue processing and staining. (**A**) Toluidine blue staining of resin-embedded semithin sections; (**B**) trichrome staining of paraffin embedded sections; (**C**) trichrome and osmium staining of paraffin embedded sections. Scale bars: 20 μm.

As mentioned before, other conditions, in addition to the counting method, have to be considered in comparing the results of fiber counting in different studies. The gold standard of tissue processing for using counting methods is toluidine blue staining of resin-embedded semithin sections (Figure 2A), which presents the dark staining of myelin sheaths due to osmium tetroxide post-fixation. Tissue processing with resin embedding better preserves nerve fiber histology than paraffin embedding. Traditional haematoxylin and eosin staining or trichrome staining of paraffin-embedded nerves do not allow us to clearly distinguish myelinated fibers (Figure 2B). Therefore, staining with osmium of paraffin-embedded nerves is a valid alternative to the conventional resin embedding-based protocol with some advantages: it is much cheaper, it can be adopted by any histological laboratory and it allows both counterstaining with haematoxylin and eosin staining or trichrome staining (Figure 2C) and the conduction of immunofluorescence analysis [22,25].

On the other hand, the traditional resin embedding-based approach remains the elective protocol if high-resolution light microscopic observation is sought, and in all those cases for which electron microscopy is also necessary.

In a few studies [26,27], myelinated nerve fibers are also counted using transmission electron microscopy. A comparative study between light and electron microscopy quantitative analysis shows that the light microscopy estimations significantly underestimate the total number of myelinated fibers compared to the evaluation made using transmission electron microscopy [27]. This discrepancy is mainly observable in regenerating nerves that contain a higher number of very small fibers and which are only detectable using electron microscopy.

Transmission electron microscopy is also very useful for a qualitative morphological analysis. For example, it allows us to follow the important process of remyelination during nerve regeneration. All phases of axon enwrapment by Schwann cells could be followed by an evaluation of the myelin structure, the number of myelin laminae and possible defects in myelin compactness [28,29]. A dysregulated remyelination could lead to a compromised functional recovery.

Moreover, transmission electron microscopy allows the analysis of the relationship between regenerating tissue and biomaterials used as prosthesis for nerve repair, the effect of their degradation or the fate of nanoparticles (e.g., iron oxide, silica) used for the delivery of factors conducive to the improvement of nerve regeneration [30].

Quantitative studies in which different fixation, embedding and staining procedures were performed cannot be compared, since the number and size of the fibers evaluated could change due to their differing ability to maintain the histological structure of the fibers (i.e., swelling after paraffin embedding) or differing recognition capacities due to the quality of the section (histology more or less maintained) or the quality of the microscope and camera used. The effects of errors generated by a different sample preparation on the quantification of myelinated fibers could be an over- or under-estimation of the fiber number and size parameters. An optimal procedure is to treat all samples of all experimental groups using the same protocol and, if possible, at the same time.

## 4. Nodal and Internodal Length Analysis

**Figure 3:**
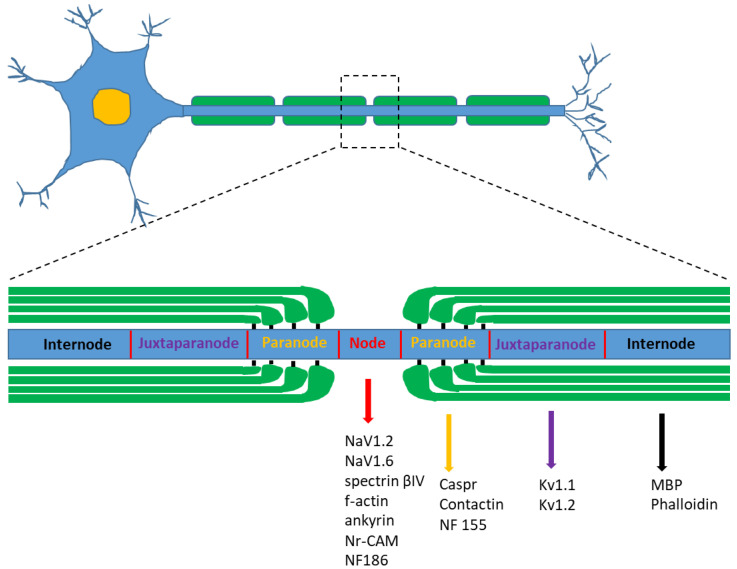
Schematic illustration showing the structure of a myelinated neuron and the different subdivisions of the axonal domain: the node of Ranvier, the paranode, the juxtaparanode and the internode. Each of these domains is characterized by the expression of specific proteins.

The internode consists of the largest domain of the myelinated fiber and is the area of compact myelin between two adjacent nodes of Ranvier [31]. The nodes are the short periodical interruptions in the myelin sheath where ion flow across the membrane occurs, resulting in the propagation of action potentials along the axon via saltatory conduction. Around the nodes, glial and axonal membranes come into intimate contact, resulting in the morphological and functional subdivision of axonal domains into node, paranode, juxtaparanode and internode (Figure 3). Each domain contains specific sets of ion channels and cell adhesion molecules that mediate axo–glial interactions and the entire axon, including its cytoskeleton, organelle content and transport machinery, is differentially organized at these sites [31,32].

The correct function of a nerve fiber depends on the integrity and correct organization of the node/internode structure. An altered organization of these domains results in conduction block and dysfunction, important sources of morbidity in disorders affecting myelin sheath. In healthy nerve fibers, internodal length is proportional to fiber diameter and is optimized during development to ensure maximal neuronal conduction velocity; during peripheral nerve regeneration, the axon–Schwann cell interactions are renewed and regenerated and remyelinated nerve fibers have thinner myelin sheaths with shorter internodal lengths, leading to slower conduction rates [33]. This, in turn, has implications for nerve function and repair. The evaluation of nodal and internodal organization thus has significant clinical implications that should be correlated with the electrophysiological method for recording nerve conduction velocity.

To measure the internodal length, it is necessary to obtain single fibers dissociated for a distance of many nodes. The internodes may be quite long, especially in control/uninjured nerves (up to few millimeters long). Nerves are usually dissociated under a dissecting microscope until single fibers are obtained. A variable number of internodes can be measured (from 100 to 300) depending on the animal model and the size of the nerve sample directly under a stereomicroscope or with a light microscope.

Once the measurement of length of individual internodes is obtained, it can be useful to plot it against that of its respective mean diameter [33].

Another method to study nodal and internodal structures, is to process the nerve samples for immunostaining analysis with appropriate antibodies (i.e., staining with phalloidin or myelin basic protein (MBP) to highlight the internodes). Node of Ranvier, paranode and juxtaparanode regions can then be detected by specific antibodies (Figure 3). Nodes are ~1 μm long gaps between myelinating Schwann cells and represent sites where the axon is exposed to and communicates with the extracellular environment. At the centre of nodal region, voltage-gated sodium channels are clustered at high density (>1200/mm^2^) and are responsible for generation and propagation of the action potential during saltatory conduction. Antibodies used to identify the region of the node include therefore anti-pan-NaV antibodies (NaV1.2, NaV1.6) [34,35]. The axonal segment underlying the node is highly specialized with a distinct submembranous cytoskeleton containing the actin-binding protein spectrin βIV, f-actin and the cytoskeletal adaptor ankyrin [36,37,38]. Additionally, adhesion molecules such as Nr-CAM and Neurofascin186 (NF186) are clustered at the node and seem to interact with glial proteins in the microvilli that project from the end of the Schwann cell to closely contact the nodal axolemma.

At the lateral end of compact myelin, the tight membrane packing is split up to form the paranode. At the contact site of the paranode, loops with the axolemma septate-like junctions are formed. On the axonal site, a complex of the adhesion molecules Caspr (contactin-associated protein, also known as paranodin) and the glycosylphosphatidylinositol (GPI)-linked cell adhesion molecule contactin accumulate at the paranode. Antibodies used to identify the region of the paranode therefore include anti-contactin-associated protein (anti-caspr) [34]. The glial membrane at the paranode region contains neurofascin 155 (NF155), a spliced isoform of the cell adhesion molecule neurofascin that is specifically found at the glial loops [39].

The juxtaparanode cannot be identified as a morphological structure but is defined as 10–15 nm wide area adjacent to the paranode. Functionally important is the clustering of delayed rectifying potassium channels, which are important for the excitability of the nodes [40]. Anti-Kv1.1 and 1.2 antibodies can therefore be used to label the juxtaparanode region of myelinated nerve fibers [34].

For node length analysis, a variable number of nodes can be selected (the criterion for selection can be that the nodes lay approximately parallel to the plane of section). Confocal images can be analyzed using different software, such as ImageJ, Reconstruct, MATLAB (The MathWorks, Inc., Natick, Massachusetts, United States) script or Imaris software [35,41]. Automated methods for the detection of the nodes of Ranvier in serial stacks of peripheral nerve images have also been proposed [42].

The nanoscale organization of nodal proteins can also be visualized by stimulated emission depletion (STED) super-resolution microscopy of teased nerve fibers [43].

Moreover, from a qualitative point of view, it is also possible to visualize node and internode structures using other techniques, such as high-resolution light microscopy of toluidine blue stained longitudinal sections [44] or electron microscopy analysis [45].

## 5. Retrograde Labelling Technique for Regenerating Fiber and Neuron Staining

Retrograde tracing techniques are neuroanatomical methods used to map connections between regions of the nervous system at distance, but they also provide a useful method for analyzing the specificity of axonal regeneration.

During nerve regeneration, the regrowing axon sprouts up to 25 daughter axons; as regeneration proceeds, most of these supernumerary axons are lost because they fail to make a connection with a peripheral target; there are, however, a higher number of axons in the regenerated distal stump compared to the proximal one several months after injury [46]. The retrograde tracing technique is useful because it allows us to count regenerating axon fibers without having the overestimation caused by axonal branching.

Retrograde tracing is based on the uptake of a fluorescent dye that is conveyed retrogradely by axonal transport to the cell body of the neuron (located in the anterior horn of the spinal cord in the case of motor neurons, or in the dorsal root ganglion in the case of sensory neurons). This tracer can be applied anywhere along the course of the nerve or directly into the target muscle or in the skin, and after a suitable time has passed to allow for the uptake and transport of the tracer, the tissue is fixed, sectioned and the retrograde tracer visualized microscopically.

Different technical issues must be considered in the use of retrograde tracers, including uptake mechanism, labelling efficiency, possible fading of the tracer, interaction with other tracers and persistence of the tracer (when using multiple tracers), potential neurotoxicity, suitable stability in vivo and compatible tissue processing for histological/immunohistochemical staining [47,48].

“Sequential tracing” is an especially useful technique used to investigate the accuracy of regeneration toward a specific nerve branch by injecting a first tracer in a target region before nerve injury to label the original neuronal population and applying a second tracer after injury and regeneration to label the regenerated axons. However, particular attention must be taken because, if the first tracer remains available in the target region, regenerated axons could uptake both the first and second tracer, compromising cell count and data interpretation [49].

Multiple tracers can be applied at the same time to different nerve branches; for example, to investigate the accuracy of motor versus sensory regeneration or to investigate dispersion of axonal collaterals originating from the same motor neuron to different branches [50].

An extensive review on the different dyes used for retrograde labelling in peripheral nerve research with pros and cons in their use has been published by Hayashi and colleague [51]. The different dyes will therefore be described hereinafter only briefly (Table 1).

Fast Blue (FB) is an aqueous tracer that primarily labels the neuronal cytoplasm producing intense blue fluorescence; it can be transported effectively over long distances and represents the tracer of choice for retrograde motor neuronal labelling in long-term experiments [52] since it has been reported to persist for at least 6 months. However, this could cause the tracer to be dispersed over time and to be re-uptaken when using FB as the first tracer in a multiple labelling study [51].

The ultraviolet excitable dye hydroxystilbamidine methanesulfonate (Fluoro-Gold (FG)) has been shown to be highly sensitive and permanently retained in retrogradely labelled neurons. The tracer extensively labels cytoplasm and dendritic processes, producing bright white fluorescence, while the nucleus remains devoid of stain. The FG can also be used for electron microscopy studies [53]. It has been described as a significant reduction of FG-stained cells after 12 weeks and has been suggested to limit the use of FG for short-term experiments due to leakage or degradation of the dye [52].

Fluoro Ruby (FR, dextran tetramethylrhodamine) is a nontoxic and relatively inert hydrophilic polysaccharide. FR produces a deep red fluorescence in the cytoplasm and proximal dendrites and has been shown to be a reliable tracer for regenerating fiber tracts [54]. However, a significant reduction in neuronal labelling after 4 and 12 weeks has been shown, suggesting its use for short-term experiments [52] or as a secondary tracer in sequential labelling studies.

Finally, Diamidino Yellow (DY) produces yellow fluorescent labelling of the neuronal nucleus. It is frequently used in conjunction with FB or FG in double-labelling experiments [47].

There is no ideal dye; the dye to be used in a certain experiment must therefore be chosen carefully, knowing the pros and cons of the different tracers and depending on the purpose of the study (e.g., short or long-term experiment, use of single or multiple tracers, the need to immunohistochemically stain other structures). Moreover, there is no optimal condition for retrograde labelling shared by the different fluorescent dyes. Every dye should follow a specific protocol (i.e., concentration of the dye, method of applying the dye) that also depends, besides the purpose of the study, on the chemical nature of the dye (hydrophilic or lipophilic).

There are different methods that can be used to apply the dye to the nerve. The most used it to place the proximal stump of the transected nerve in a cup containing a solution with the dye for 1 or 2 h. The injection of the dye directly in the target muscle by a small-gauge needle is also a widely used method, even if the number of neurons that are labelled is less than with the previous method. The dye solution can also be injected directly into the nerve with the use of a Hamilton syringe.

The neuron counting is, probably, the most difficult part during experiments with retrograde tracers. To date, most of the researchers use stereology to obtain unbiased estimation of labelled cells [51]. An innovative method called retroDISCO, that optically clears whole mouse spinal cord and allows us to count motor neurons without the need for histological sectioning, thus avoiding double counting of cells, has also been described [55].

To conclude, an important advantage of retrograde tracing techniques is thus the possibility of quantifying the specificity of nerve regeneration and to avoid overestimation due to axonal branching. However, methodological issues can be encountered, and interpretation of the results is not always easy.

In recent years, the development of viral tracers has rapidly grown up, with the advantage of targeting transgene expression in specific neuronal types using genetic strategies or recombinant viral technology [56,57,58].

## 6. Immunohistochemistry for Identifying Proteins Specific for the Different Cellular Components of the Peripheral Nerve

Immunohistochemistry (IHC) is a technique that allow the visualization of cellular and molecular components in tissues or cells. This capability is achieved through combinations of specific antibodies tagged with fluorophores or enzymes. Consequently, its applications in the nerve regeneration field are numerous and a variety of experimental conditions can be employed [59].

A preliminary essential step in IHC staining is the fixation of the sample in order to preserve the good histology of the tissue and, at the same time, to maintain its antigenicity. Indeed, an “ideal” fixative promotes the immobilization of target antigens maintaining cellular and tissue architecture in order to allow antibodies to reach targeted components. Following fixation, and before any antibody incubation, blocking must be performed to prevent wrong binding to non-target epitopes. A primary antibody labels a protein and then a secondary antibody binds oneself to the primary one. In immunofluorescence protocol, the secondary antibody is joined with a fluorochrome, while, in immunoperoxidase staining, the antibody is bounded to peroxidase enzyme, which catalyzes a reaction in which the target protein is specifically stained in brown [59].

**Figure 4:**
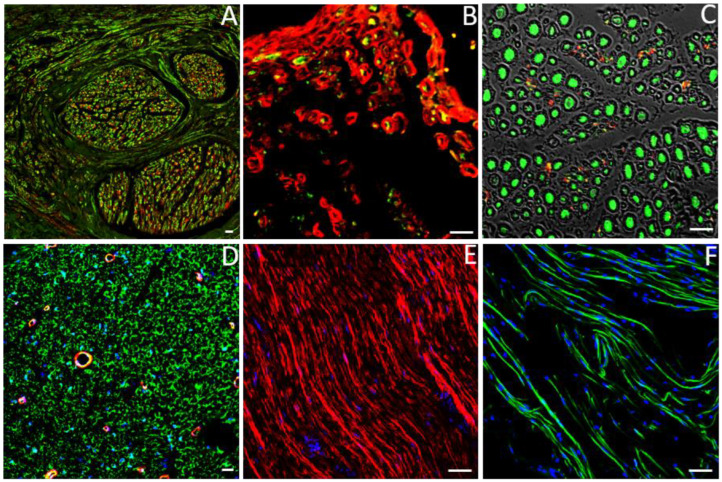
Representative transversal (**A**–**D**) and longitudinal (**E**,**F**) sections showing immunolabeled nerves. (**A**) transversal cross section of rat median nerve in which axons are labelled with anti NF-200 (green) and Schwann cells are identified with anti S100β (red); (**B**) high magnification of a transversal section showing axons labelled with anti NF-200 (green) and Schwann cells with anti S100β (red); (**C**) high magnification of a nerve cross section immunolabeled with anti- NF-200 (green) and anti-peripherin (red); (**D**) transversal section immunolabeled with anti S100β (green), anti-SMA (Smooth Muscle Actin, red, for vessels), while nuclei are detectable by DAPI (blue); (**E**) longitudinal section showing nerve fiber labelled with anti S100 (red) and DAPI (blue); (**F**) longitudinal section showing axons labelled with anti βIII tubulin (green) and DAPI (blue). Scale bar: 20 μm.

Focusing on peripheral nerve injury, IHC is one of the most widely used methods to study the degree of the regenerative process thanks to the variety of specific available antibodies that allow us to detect axons, Schwann cells, macrophages, fibroblast, vessels and other nerve regeneration elements (Figure 4) [60,61].

The aim of using IHC on a nerve sample is twofold: (i) identifying a particular cell type using specific cell markers and (ii) verifying if a particular cell type expresses the protein of interest. This first application of IHC could be useful for understanding cellular behavior during peripheral nerve regeneration process. For example, the evaluation of the interaction between Schwann cells, endothelial cells and regenerating axons allows us to demonstrate that blood vessels provided a path for Schwann cell migration during nerve fiber regeneration [62]. For this aim, it is important to know which of the proteins expressed by the cells of interest; for example, for identifying the two main components of nerve fibers: axons and Schwann cells. In particular, axons could be labelled with several markers, such as Neurofilament-200 (NF-200), an intermediate filament protein that is the key constituents of the neuronal cytoskeleton [63], βIII tubulin, a key cytoskeletal component expressed almost exclusively within neurons and axons [64]; Peripherin, a type III intermediate filament expressed mainly in small-size nerve axons [65]; Growth Associated Protein 43 (GAP43), the major component of “growth cones”, forming the tips of elongating axons, expressed by developing and regenerating neurons [66]; and Protein Gene Product 9.5 (PGP 9.5), also known as Ubiquitin Carboxyl-Terminal Hydrolase-1 (UCHL-1), a neuron specific protein expressed by neurons and nerve fibers of the central and peripheral nervous system [67].

As regards Schwann cells, depending on their phase of the regenerative process, they could be labelled with different markers such as Glial Fibrillary Acidic Protein (GFAP), a member of the type III intermediate filament protein family expressed by non-myelinating Schwann cells [68]; Myelin Basic Protein (MBP), a myelin protein used to study myelination [69]; and S100 calcium-binding protein β (S100 β), a calcium-binding protein and the most typical marker for adult Schwann cells [70]. The main antibodies used to study nerve regeneration are summarized in Table 2.

The second possible application of IHC is for demonstrating which cells express a specific protein. For example, vascular endothelial growth factor (VEGF), that represents one of the main factors involved in angiogenesis, was recently studied during nerve regeneration process to evaluate the involvement of this factor and its receptors and co-receptors during the different steps of nerve repair. Interestingly, IHC staining allowed to detect a glial localization of VEGF and VEGF receptor 2 after peripheral nerve crush injury, suggesting a potential autocrine pathway on Schwann cells [71].

However, some limitations must be considered in employing IHC: different fixation methods can often explain different immunohistochemical results; depending on the antibody used, specific conditions should be addressed to ensure that the antibodies work properly, such as set temperature, humidity and the unmasking of antigen before primary antibody incubation. Furthermore, appropriate positive and negative controls should be determined to rule out fake results [18].

Focusing on regenerated peripheral nerves, IHC could be basically employed on longitudinal and transversal cross sections. Longitudinal sections allow us to identify the site of nerve injury, to evaluate the migration of Schwann cells and the progression of regenerating axons from the proximal toward the distal portion of the nerve. In this case, attention must be taken considering that regenerating axons do not travel in a straight direction throughout the nerve, but rather in a wavy manner. Therefore, the same axon may not be seen on corresponding slices, although it may appear on the following. For this reason, longitudinal serial sections should be considered for this type of analysis.

Transversal section provides the evaluation of the total area of the nerve and could be useful to evaluate the histological features of a regenerated nerve and the outcome of the regeneration (fibers fasciculation, connective tissue distribution, myelin sheath formation, and fibers size).

IHC could be an appropriate method to identify and differentiate regeneration of motor and sensory fibers because it can be carried out combining multiple markers, even if it must be considered that the efficacy/accuracy of specific markers for motor and sensory nerve fibers remains unclear [72].

A drawback of immunolabelling is that thin sections are needed for antibody labelling and detection, and several sections need to be analyzed to get a 3D overview. Therefore, to obtain a 3D reconstruction of the sample, whole (and thick) tissues should be analyzed. However, signals cannot be detected within a non-transparent sample and it is difficult for antibodies to penetrate deeply within a tissue. To overcome these issues, Renier et al., set up “iDISCO” technology, a simple and inexpensive method that combines the clearing of tissues with antibody labelling, allowing the visualization of whole tissues in a three-dimensional way. Different approaches have been developed to optimize iDISCO methodology to make biological tissue transparent with a good efficiency and to support the immunolabelling procedure with a wide variety of antigens. Several methods have been tested including the use of non-ionic detergents, as well as dehydration-rehydration cycles using organic solvents, which remove membrane lipids, and incubation in hydrogen peroxide or methanol to stop the tissue autofluorescence [73]. Currently, iDISCO is used to study innervation and vascularization, cell proliferation and neuronal activity on whole embryos [73], but its application could be easily extended to a variety of structures and conditions. Indeed, Huesing and colleagues performed iDISCO to provide a comprehensive evaluation of preganglionic and postganglionic sympathetic innervation in mice [74]. Thanks to these suitable features, iDISCO could be easily applied to study axonal projection of motor and sensory fibers after peripheral nerve injury, allowing us to identify the pathways of regenerated nerve fibers and their connections to both central and peripheral nervous system in a three-dimensional way.

Finally, IHC can be also performed for semi-quantitative or quantitative analysis on regenerated nerve fibers even if, for a proper quantification, many aspects must be taken into account such as fixation, embedding, cutting, staining and image acquisition, that must be performed using the same conditions among samples. Many methods of quantification are available for both quantifying nerve fiber number and protein expression. Care must be taken in comparing results of studies that use different methods [75].

## 7. Fluorescent Transgenic Animal Models Specific for Nerve Fiber Analysis

The recombinant DNA technology has made it possible to significantly improve the study of the physiologic or pathologic phenomena in the nervous system, with the use of transgenic animal models expressing fluorescent reporters (Table 3).

Thy1 (CD90) represents the election protein to investigate morphologically the nervous system using transgenic models. It is a cell surface glycoprotein that contributes to intercellular communication, particularly in the immune and nervous systems. Various studies have demonstrated that Thy1 expression occurs predominantly in the late stages of postnatal development; hence, the Thy1-YFP reporter line can be a tool for studying non-embryonic neural development and response to injury. Nevertheless, it is necessary to be aware that Thy1 (CD90) labels also nerve fibroblasts [76], which could give a background signal and/or affect the result interpretation.

Mohan and colleagues [77] used a mouse model in which *Thy*1 promoter drove expression of YFP and provided a stain-free axon visualization for low-cost screening of the performance of novel bioengineered nerve guidance conduits. They commented on the importance of the use of such transgenic models, B6.Cg-Tg (Thy1-YFP) 16Jrs/J mice, in studies in which different biomaterials and conduits are compared in the promotion of the regeneration of the injured peripheral nerve. Moreover, quantitative methods could be also used for the evaluation of the axonal sprouting across the conduits by evaluating integrated pixel-intensity profiles.

Using the same Thy1-YFP transgenic mice model, Tu and colleagues [78] used an in vivo and in live monitoring system in which the nerve fibers were mapped during their regrowth, until reaching the target. For following nerve fiber regeneration, a fluorescent stereomicroscope was used and the number of branches of proper nerves was manually counted. The expression of YFP selectively in the nervous system allowed visualization and quantification of nerves.

**Table 3:** Transgenic animal models for the morphological analysis of nerve fibers.

Transgenic Animal Models	Fluorescent Structures	Aim of the Study	References
B6.Cg-Tg (Thy1-YFP) 16Jrs/J mice	YFP is expressed under the control of *Thy1* promoter	- axon visualization - in vivo and in live monitoring system of axon regeneration - quantitative evaluation of the axonal sprouting	Mohan and colleagues [77] Tu and colleagues [78]
Thy1-YFP-H mice	YFP is expressed under the control of neurospecific regulatory elements of *Thy1* gene	- mapping of the motor units - assessing the accurate reconstitution of the original motor units during the regenerative phase by axons	Nguyen and colleagues [79]
Thy1-CFP/S100β-GFP/mice	Expression of cyan fluorescent protein (CFP) in axons under the control of *Thy1* promoter and of GFP in Schwann cells under the control of *S100β* promoter	- assessing the rate of motor endplate reinnervation over time - assessing the number of terminal branches contacting a given motor endplate - assessing the number of terminal Schwann cells expressing S100β - assessing the intensity of S100β expression before and after injury	Magill and colleagues [80]
Thy1-GFP/rats	GFP is expressed under the control of *Thy1* promoter	- assessing nerve regeneration and muscle reinnervation by direct visualization	Moore and colleagues [81] Kemp et al. [82]
“Kosmos” mice	EGFP is expressed under the control of human *S100β* regulatory sequences	- labelling of SC at the intramuscular nerves and at the neuromuscular junctions	Zuo, Y., et al. [83]

The use of transgenic models also allow us to visualize muscle fibers that are innervated by single motor axons before and after reinnervation. Nguyen and colleagues [79] used Thy1-YFP-H mice, where the yellow spectral variant (YFP) of GFP was expressed under the control of regulatory elements from the *Thy1* gene. In this mouse model, up to five motor axons were fluorescent in each of several skeletal muscles to accurately reconstitute the original motor units during the regenerative phase in which axons recognized their original target. Following the mapping of the motor units, the nerve fascicles were crushed and regeneration was verified through acquisition with low-light fluorescence, confirming that the vast majority of neuromuscular junctions were re-identified and motor axons selectively reinnervated their original postsynaptic target cells.

A second type of transgenic model, Thy1-CFP/S100β-GFP, allows the visualizing in live of the reinnervation field of skeletal muscle following a crush nerve damage, with a focus on both neurons and Schwann cells (SCs). In fact, this transgenic model expresses cyan fluorescent protein (CFP) in axons under the control of *Thy1* promoter and GFP in SCs, under the control of *S100β* promoter [80]. The study of Magill and colleagues made it possible to determine (1) the rate of motor endplate reinnervation over time, (2) the number of terminal branches contacting a given motor endplate, (3) the number of terminal SCs expressing S100β and (4) the intensity of S100β expression before and after injury. This study allowed to establish quantitatively that, after nerve damage, 1:1 terminal axon to motor endplate ratio is established within 6 weeks, and that this nerve regeneration is accompanied by a progressive morphological change of Schwann cells, which stabilize at the same endpoint.

Moore and colleagues [81] developed a rat model in which the *Thy1* promoter drives neuron-specific expression of GFP to overcome significant limitations of the mice model, including small caliber nerves, nerve gap length restrictions (typically less than 1 cm in the hindlimb) and difficulty with functional and behavioral hindlimb based metrics. Different types of lesions followed by repair were tested to establish the model: nerve crush, end-to-end lesion and isograft (obtained from Thy1-GFP negative litter mates). At established endpoints the animals were anesthetized and a direct visualization of nerve regeneration and muscle reinnervation was performed, demonstrating that the Thy1-GFP transgenic model allows the nerve to be imaged multiple times, limiting the number of animals required because each animal data can be obtained at multiple time points.

A subsequent work, starting from Moore’s observations, deepened the studies on Thy-GFP transgenic rats to understand if these transgenic models show differences with wild type rats in terms of number of motor and sensory neurons, functional recovery capacity following injury, strength and muscle reinnervation and the morphological parameters of regenerated fibers [82]. These analyses demonstrated that Thy-GFP transgenic rats represent an excellent model for the study of nerve damage, repair and regeneration, maintaining the same nerve architecture and functional and morphometric recovery abilities of wild type animals.

“Kosmos” transgenic mice, expressing EGFP under the control of *S100β* regulatory sequences, were developed to enable the vital observation of glia at the neuromuscular junction [83]. However, considerable variegation in transgene expression among the transgenic lines was found. In some lines, cells in which the S100β protein is not normally found were clearly EGFP-positive; for example, their expression was detectable in spinal motor neurons. Such variegation is not unusual in transgenic animals; it must be taken into consideration and deserves an in-depth study of each experimental model. It might be attributed to variability in the site of transgene integration and methylation as well as the absence in the transgene construct of some genomic sequences normally conferring control of the expression of the endogenous gene. The field of nerve fiber research can take advantage of the use of fluorescent transgenic animals which are able, through the selective visualization of only some cell populations, to trace axonal regrowth following injury, the behavior of the glial component in the various regenerative phases or the reinnervation of muscle fibers. The major advantage of this method is the possibility of observing all these important aspects in live mode without sacrificing the animals, saving several endpoints and staining methods.

However, variability among animal subjects also represents a significant limitation, making a deep validation within animal groups necessary; indeed, it is essential. The key point in the research that exploits fluorescent transgenic animals is undoubtedly the validation of the models and the careful comparison with wild type animals in order always to guarantee the credibility of the data provided.

## 8. Conclusions

Objective, standardized and reliable evaluation methods are essential to properly assess the degree of peripheral nerve regeneration. There are several outcome measures that have been and are currently used in preclinical studies [84,85,86,87].

Morphological parameters are the most used predictors of peripheral nerve regeneration and the analysis of regenerating fibers is one of the most frequently analyzed outcomes that usually mirrors the degree of nerve regeneration. In this review, we therefore provided an overview of the most used morphological assessment methods that researchers employed in studies on the regeneration of peripheral nerves. It is not possible to indicate which is the best method because it depends on many factors, including the scientific goals of the research, and each method can be used to assess only a part of the outcome of the regenerative process. An appropriate combination of different evaluation methods is therefore always preferred.

It is important to carefully plan the study design and to decide in advance analyses to be performed, because the different morphological methods require specific tissue processing. Moreover, knowing pros and cons of the different methods should make the choice more conscious (Table 4).

Research on peripheral nerve regeneration is a constant challenge in the field of regenerative medicine. There are many research groups around the world who are interested in this field of research, with the common goal of improving the quality of life of patients suffering from peripheral nerve injuries. In preclinical research, in order to identify the best strategies to improve nerve regeneration, it is essential to use standardized protocols in order to be able to compare the results deriving from different studies. At the moment, there is still a lack of standardization, especially in the use of morphological methods, which nonetheless remain among the most widely used methods to evaluate the degree of regeneration in preclinical research. When analyzing and comparing different results, it is therefore necessary to understand whether the methods used are effective, reliable and suitable for the proper purpose of the research; otherwise, the interpretation of the data could lead to wrong conclusions.

Finally, all these morphological methods are applied following animal sacrifice—or, if in live view, on transgenic animals—and are therefore not applicable on humans. In view of a clinical translation, in vivo morphological analyses should also be considered in order to monitor, step by step, the regeneration phases in humans. Unfortunately, so far, most technologies are only experimental, with poor translation to the clinic [88].

## Figures and Tables

**Table 1:** Retrograde dyes with their excitation and emission wavelengths.

Name	Excitation	Emission
Fast Blue (FB)	365	420
Diamidino Yellow (DY)	365	>500
True Blue (TB)	365	405
Granular Blue (GB)	365	410

**Table 2:** Axonal and glial antibodies useful to study nerve regeneration.

Antibody	Role	Target	References
NF-200	Intermediate filament protein of neuronal cytoskeleton	Axons	Kriz and colleagues [63]
βIII tubulin	Cytoskeletal component expressed almost exclusively by neurons and axons	Axons and neurons	Hausrat and colleagues [64]
Peripherin	Type III intermediate filament protein	Small-sized axons	Fornaro and colleagues [65]
GAP43	Major component of “growth cones” of elongating axons	Developing and regenerating neurons	Strittmatter and colleagues [66]
PGP 9.5/UCHL-1	Neuronal specific protein member of ubiquitin carboxyl-terminal hydrolase domain	Neurons and nerve fibers	Romeo and colleagues [67]
GFAP	Type III intermediate filament protein	Non-myelinating Schwann cells	Yang and colleagues [68]
MBP	Major constituents of myelin sheath of Schwann cells	Myelinating Schwann cells	Smith-Slatas and colleagues [69]
S100 β	Calcium-binding protein	Adult Schwann cells	Morton and colleagues [70]

**Table 4:** Morphological methods for peripheral nerve regeneration analysis.

Morphological Methods	Type of Analysis	Limits of the Technique	References
Histological Staining	- Qualitative analysis of nerve tissue - Quantitative analysis of myelinated nerve fibers	- Bias for quantitative analysis using different types of fixation, embedding, staining	Raimondo and colleagues [12] - Carriel and colleagues [13] - Carriel and colleagues [14] - Di Scipio and colleagues [25]
Transmission Electron Microscopy	- Ultrastructural analysis - Qualitative and quantitative analysis of myelinated and unmyelinated nerve fibers - Analysis of myelination phases - Analysis of the interaction of graft materials with regenerating tissue - Visualization of delivered-nanoparticle fate	- Complex, long and expensive analysis - Toxicity of reagents (fixative, osmium, propylene oxide)	- Soltanpour and colleagues [26] - Ronchi and colleagues [27] - Salzer and colleagues [28] - Liu and colleagues [29] - Ziv-Polat and colleagues [30]
Retrograde Labeling	- Map connection between distant regions of the nervous system - Specificity of axonal regeneration (avoids overestimation caused by axonal branching) - Retrograde identification of neuronal bodies (to discriminate between motor and sensitive regenerating neurons)	- Interpretation of the results is not always easy - Possible technical issues (uptake mechanism, labelling efficiency, possible fading of the tracer, interaction with other tracers and persistence of the tracer when using multiple tracers) - Potential neurotoxicity - Not always suitable stability in vivo	- Puigdellívol-Sánchez and colleagues [47] - Mi and colleagues [48] - Puigdellıvol-Sánchez and colleagues [49] - de Ruiter and colleagues [50] - Hayashi and colleagues [51] - Novikova and colleagues [52] - Schmued and colleagues [53] - Radtke and colleagues [54] - Žygelytė and colleagues [55] - Xu and colleagues [56] - Liu and colleagues [57] - Qiu and colleagues [58]
Immunohistochemistry Analysis	- Identification of specific cell types using specific cell markers - Evaluation of the expression of the protein of interest in a particular cell type	- Antibody validation needed - Bias of antibody-efficacy using different types of fixation, embedding, staining	- Im and colleagues [59] - Verdu and colleagues [61] - Fornar and colleagues [65] - Strittmatter and colleagues [66] - Romeo and colleagues [67] - Yang and colleagues [68] - Smith-Slatas and colleagues [69]
Transgenic Models	- Observation of the cytoarchitecture of the regenerating nerve - Observation of different phases of regeneration in live mode, without sacrificing the animals, saving several endpoints and staining methods	- Complex validation of animal models - Possible high variability among animal subjects	- Tu and colleagues [78] - Nguyen and colleagues [79] - Magill and colleagues [80] - Moore and colleagues [81] - Kemp and colleagues [82] - Zuo and colleagues [83]

## Data Availability

Not applicable.
